# Microfluidic DNA-based potassium nanosensors for improved dialysis treatment

**DOI:** 10.1186/s12938-019-0692-8

**Published:** 2019-06-11

**Authors:** Alexander F. Smith, Bin Zhao, Mingxu You, Juan M. Jiménez

**Affiliations:** 1 Department of Mechanical & Industrial Engineering, University of Massachusetts Amherst, Amherst, MA 01003 USA; 2 Department of Chemistry, University of Massachusetts Amherst, Amherst, MA 01003 USA

**Keywords:** Potassium sensor, DNA nanosensor, G-quadruplex, Microfluidics, Fluorescence oligonucleotide

## Abstract

**Background:**

Patients with end-stage renal disease (ESRD) have failed kidney function, and often must be treated with hemodialysis to extend the patient’s life by artificially removing excess fluid and toxins from the blood. However, life-threatening treatment complications can occur because hemodialysis protocols are adjusted infrequently, as opposed to the kidneys which filter blood continuously. Infrequent blood tests, about once per month on average, are used to adjust hemodialysis protocols and as a result, patients can experience electrolyte imbalances, which can contribute to premature patient deaths from treatment complications, such as sudden cardiac death. Since hemodialysis can lead to blood loss, drawing additional blood for tests to assess the patient’s kidney function and blood markers is limited. However, sampling multiple drops of blood per session using a microfluidic device has the potential to reduce not only the amount of blood drawn and avoid unnecessary venipuncture, but also reduce costs by limiting medical complications of hemodialysis and provide a more comprehensive assessment of the patient’s health status in real time.

**Result:**

We present preliminary proof-of-concept results of a microfluidic device which uses DNA-based fluorescence nanosensors to measure potassium concentration in a flowing solution. In a matter of minutes, the flowing potassium solution reduced the fluorescence intensity of the nanosensors to a steady-state value.

**Conclusions:**

These proof-of-concept results demonstrate the ability of our DNA-based nanosensors to measure potassium concentration in a microfluidic device. The long-term goal is to integrate this technology with a device to measure potassium and eventually other blood contents multiple times throughout a hemodialysis session, enabling protocol adjustment similar to a healthy kidney.

## Background

In the USA alone, more than 726,000 patients suffer from kidney failure with more than 124,000 new yearly diagnoses and 28% mortality rate [1]. More than 71% of kidney failure patients require frequent hemodialysis to filter their blood artificially, and without dialysis the patient median survival time is only several weeks [2]. The goal of hemodialysis is to remove waste from the blood and maintain equilibrium of body fluids, functions naturally performed by the kidneys. During hemodialysis sessions, the patient’s blood is pumped from their arm into a machine which uses a dialyzer to filter the blood before it is infused back into the patient. The dialyzer contains two chambers, one for blood and one for dialysate, a solution of water, electrolytes, and salts. Toxins from the blood are diffusively transported across a semi-permeable membrane into the dialysate to provide artificial filtration that replaces the function of the kidneys.

In contrast to the kidneys, which function and adjust electrolytes continuously, adjustment of hemodialysis protocols and the dialysate is dependent on each dialysis center. Dialysates are adjusted infrequently and often prepared generically using a uniform concentration of electrolytes without consideration of each individual patient’s blood electrolyte concentration. Blood electrolyte concentrations can be assessed by blood tests; however, these are costly, carry risks such as blood loss, anemia, hematoma, and infection, and can be impractical if required for each of the multiple weekly dialysis sessions [3–6]. Consequently, electrolyte levels, including potassium, are assessed infrequently and can contribute to blood electrolyte imbalances [7–9]. The leading cause of mortality in hemodialysis patients is sudden death due to cardiac arrest, which accounts for 45% of deaths [10–13]. Since potassium is the primary ion regulating cardiac repolarization, potassium imbalances have been hypothesized to trigger arrhythmias and contribute to sudden cardiac arrest in hemodialysis patients [11, 14]. Hence, monitoring potassium levels in hemodialysis patients is paramount.

Presently, blood electrolyte levels of dialysis patients are assessed with UV absorbance [15], optical [16, 17], or electrochemical [18–20] based assays that require large blood samples. Results commonly require 24 h or longer before they are received. More recently, molecular fluorescence-based techniques have been explored in research laboratory settings to measure potassium in solution [21, 22]. Molecular fluorescence-based assays have the potential of higher specificity and sensitivity than the more popular electrochemical (potentiometric) and electrical-impedance methods [23]. Advances in molecular sensing techniques have led to the development of novel DNA-based oligonucleotide nanosensors that can measure cations using G-quadruplex structures, where four guanines in a DNA strand encapsulate a monovalent ion in a plane [21]. While the physiological function of G-quadruplex structures is likely involved in DNA replication, transcription, and repair [24], with the addition of fluorescence dyes to the end of the GGGG sites, direct fluorescence measurement of electrolytes is possible [21]. G-quadruplex has been previously used for potassium ion detection. More recently, a selective potassium ion G-quadruplex sequence has been identified, with little interference from competitive ions at physiological concentrations [22].

In this study, based on the selective G-quadruplex sequence, we developed a first-generation microfluidic-based DNA nanosensor to measure potassium in an aqueous solution with the ultimate goal of measuring electrolytes in blood plasma at the point of care. In the device, fluorescence and quenching dyes FAM and DABCYL, respectively, are integrated into scaffold oligonucleotides rendering the DNA nanosensor. The DNA nanosensor is immobilized on the glass bottom of the device. Upon excitation, the absence of potassium results in light emission, while interaction between potassium and the DNA nanosensor leads to quenching of the fluorophore. Using the nanosensor, measuring serum electrolyte levels with a small sample volume in a point-of-care microfluidic device presents an opportunity to reduce clinical errors that can occur prior to sample analysis due to labeling, collection, and transport mishaps [25], in addition to reducing the amount of blood loss experienced by the patient. Furthermore, a microfluidic device allows for more frequent monitoring of a patient’s electrolyte levels by measuring multiple drops of blood per hemodialysis session, providing a more comprehensive assessment of the patient’s health.

## Methods

### Preparation of oligonucleotide sample for DNA nanosensor

**Fig. 1:**
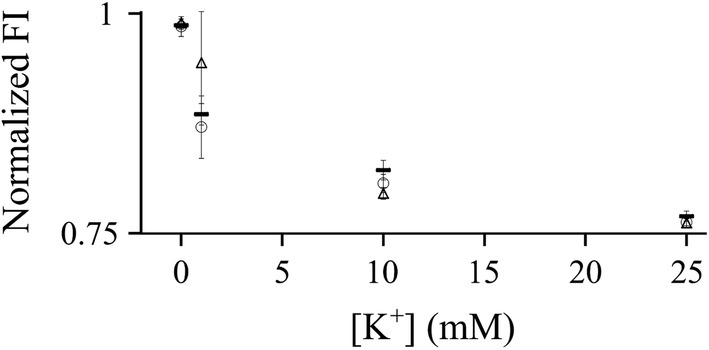
Steady-state fluorescence intensity (FI) response to K^+^ was measured as a function of the magnesium concentration in the buffer used to hybridize DNA nanosensors. Fluorescence intensity was normalized with respect to the DNA nanosensor fluorescence signal in the presence of the control buffer (PB + MgCl_2_), without K^+^. To optimize the concentration of magnesium in buffer, K^+^ was added at concentrations of 0, 1, 10, and 25 mM to 1 µM DNA nanosensor samples, each prepared with a buffer containing a different magnesium concentration: 0.85 mM (large circle), 1.05 mM (white up-pointing triangle), and 5 mM (figure dash)

The DNA nanosensor, composed of three hybridized oligonucleotides, was prepared by mixing 25 µM fluorescence-emitting oligonucleotide (FAM), 25 µM fluorescence-quenching oligonucleotide (4-((4-(dimethylamino)phenyl)azo)benzoic acid, DABCYL) and 25 µM oligonucleotide scaffold molecule in 0.1 M phosphate buffer containing 0.85 mM MgCl_2_ (PB + MgCl_2_). Varying magnesium concentration in the buffer solution during hybridization did not affect sensor affinity for K^+^ (Fig. 1). The sample solution was heated for 5 min at 95 °C to allow for annealing, and then stored for at least 15 min at room temperature to allow for DNA hybridization. The completed DNA-based sensor was analyzed for its sensing ability at room temperature but was stored at − 20 °C when not in use to keep the oligonucleotide structure stable. The formation of the DNA probe was characterized with a 16% native polyacrylamide gel electrophoresis (PAGE). The gel was run in 1× TBE under 100 volts for 30 min and imaged with Blue View transilluminator (Vernier) directly without staining.

### Fluorescence spectrum measurement

A static fluorescence assay was performed with a fluorescence spectrometer (HORIBA, PTI fluorescence system) to test the function of the DNA nanosensor for K^+^ sensing at room temperature. The DNA nanosensor at a concentration of 1 µM was used and K^+^ was added at concentrations of 0, 1 μM, 1 mM, and 10 mM to measure the change in fluorescence intensity. Potassium chloride (KCl) was the source of K^+^ for all experiments.

### DNA nanosensor concentration sensitivity

A DNA nanosensor concentration assay was performed with a fluorescence spectrometer (HORIBA, PTI fluorescence system) in real time to further analyze the relationship of the DNA nanosensor concentration for constant K^+^ concentrations at room temperature. The DNA nanosensor concentrations of 0, 1, 5, 10, 16.6 and 33.3 µM were used as K^+^ was added sequentially. K^+^ concentrations investigated were 0, 1, 5, and 25 mM.

### DNA nanosensor ion selectivity measurement

To enhance the selectivity of the nanosensor, EGTA (ethylene glycol-bis(β-aminoethyl ether)-*N*,*N*,*N*′,*N*′-tetraacetic acid) at a concentration of 3 mM was added to samples containing K^+^, Na^+^, and Ca^2+^ and incubated at room temperature for 15 min to abrogate the interference of calcium ions [26]. The 0.5 μM DNA nanosensor solution was then added and incubated for 2 h at room temperature, followed by fluorescent measurements. A fluorescence spectrometer was used to measure the fluorescence intensity of samples with and without EGTA. Samples were diluted by a factor of 10 and 100 times from physiological concentrations.

### Surface treatment of glass slides

Standard 75-mm × 38-mm glass slides (Thermo Fisher Scientific) were immersed in ethanol for 15 min for cleaning and dried with filtered nitrogen gas. The slides were exposed to oxygen plasma (Harrick Plasma, Plasmaflo) for 1 min to create OH^−^ groups on the glass surface. The modified slides were immediately immersed in a 1% v/v (3-glycidyloxypropyl)trimethoxysilane solution for 20 min to allow the epoxy-silane to bond to the OH^−^ groups on the slides. The treated glass slides were washed using deionized water, and dried quickly using filtered nitrogen gas.

### DNA nanosensor concentration sensitivity on glass slide

DNA nanosensors were coated at different concentrations of 0, 0.1, 1, 5, 10, 15, 20, and 25 µM on treated glass slides. The DNA nanosensor fluorescence signal was measured at 37 °C using epifluorescence microscopy. Images were acquired immediately after coating and the fluorescence intensity for each concentration was determined using ImageJ software (NIH, Bethesda, MD).

### Fabrication of DNA-based nanosensor microfluidic device

The microfluidic device was fabricated using standard photolithography and soft lithography protocols. A master mold with a design of five parallel microfluidic channels was created using a silicon wafer. Polydimethylsiloxane (PDMS), mixed with a base/agent ratio of 10:1, was poured to completely cover the device mold. The PDMS was cured for 24 h at room temperature followed by 24 h at 65 °C to minimize the effects of PDMS shrinking. The PDMS device was cut from the mold using a razor blade. The PDMS device was then bonded to the silane-treated glass slides by exposing the PDMS device and treated glass slide to oxygen plasma for 1 min in a plasma cleaner (Harrick Plasma, Plasmaflo), pressing the PDMS device onto the treated glass slides with even pressure, and heating the assembled device at 65 °C for 30 min.

The device channels were filled with a 25 µM amino-modified DNA nanosensor solution using a syringe and incubated overnight at room temperature to allow the DNA nanosensors to immobilize through amine–epoxy binding [27]. Afterwards, the channels were washed ten times with buffer solution (PB + MgCl_2_) to remove excess unbonded oligonucleotides. The completed microfluidic device, functionalized with the DNA nanosensors, was analyzed at room temperature and stored at 4 °C until used. A syringe pump (PHD Ultra, Harvard Apparatus) was connected to the device inlet and infused at a rate of 0.02 mL/h and yielded a shear rate of 91 s^−1^. These experiments were conducted at 37 °C and a minimum of three samples were analyzed for statistical significance.

## Results

### DNA nanosensor formation

**Fig. 2:**
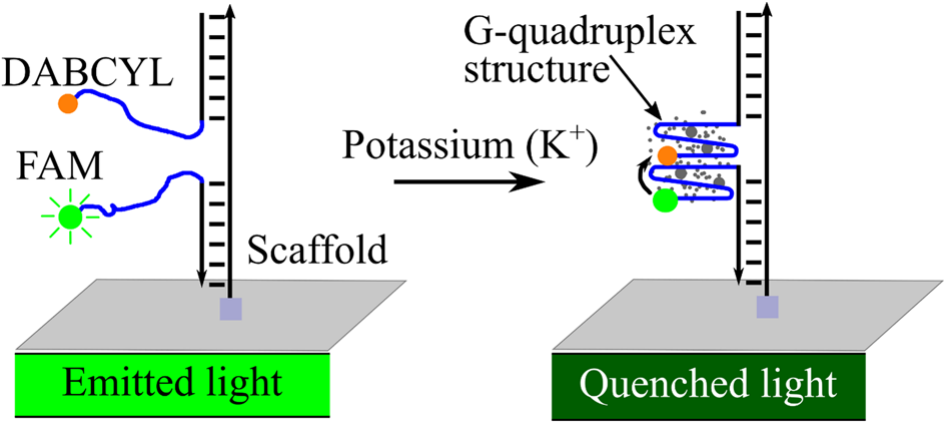
The DNA nanosensors are composed of three hybridized oligonucleotides: a fluorophore (FAM), a quencher (DABCYL), and a scaffold oligonucleotide. In the presence of buffer, the DNA nanosensor complex fluoresces. When potassium is present, the DNA nanosensor complex forms a more compact G-quadruplex structure and brings the fluorophore and quencher closer together, causing the fluorescence signal to decrease

**Fig. 3:**
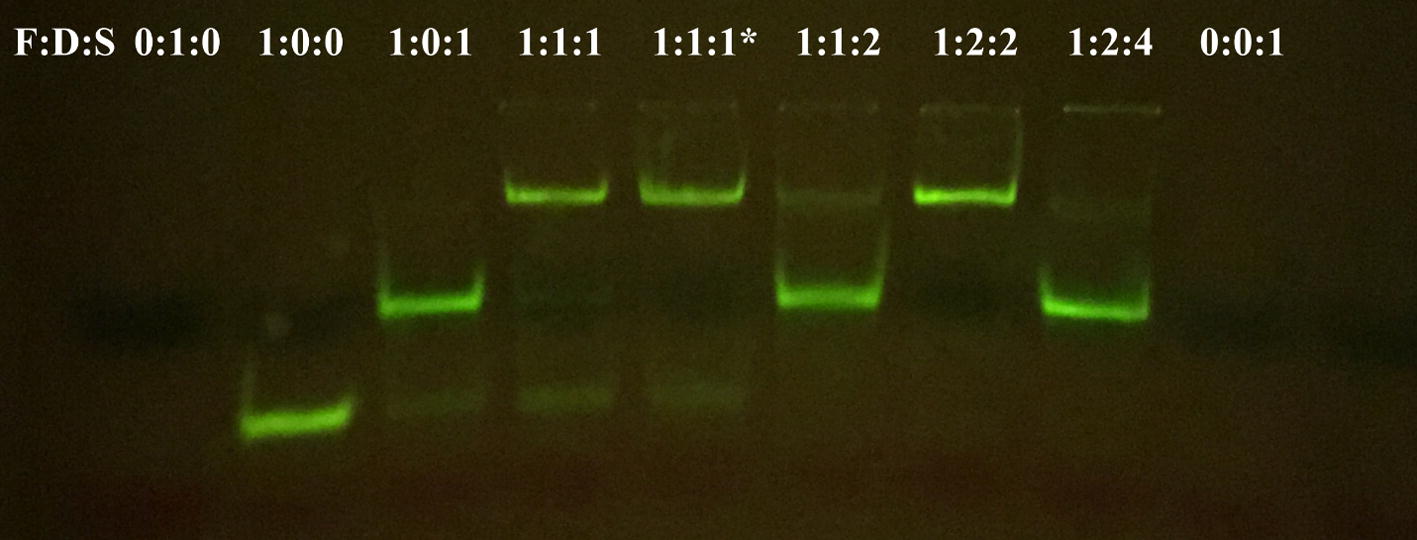
PAGE gel results for different combinations and different molar ratios of the three oligonucleotides that make up the DNA nanosensor complex. Each well differs by the molar ratio of FAM:DABCYL:scaffold

In these experiments, fluorescence and quenching dyes FAM and DABCYL, respectively, are hybridized with scaffold oligonucleotides that provide a structure for the DNA nanosensor. In the absence of potassium, the fluorescence dye structure emits light, while it is quenched in the presence of potassium (Fig. 2). In our system, sensing the presence of K^+^ depends on proper hybridization of the three oligonucleotides. We used PAGE gels to confirm stable hybridization of the fluorescence-emitting oligonucleotide (FAM), fluorescence-quenching oligonucleotide (DABCYL), and oligonucleotide scaffold molecule. To do this, we assessed the size of the structures of nine oligonucleotide samples, each containing one, two, or all three of the oligonucleotides (FAM, DABCYL, scaffold) used to form the K^+^ DNA nanosensors. Different molar ratios of the three oligonucleotides were tested. The samples were hybridized at a temperature of 95 °C for 5 min and incubated at room temperature for 15 min before they were run through the PAGE gel for 30 min. After being run through the gel, the hybridized structure, composed of all three oligonucleotides, moved the least through the PAGE gel relative to individual oligonucleotides and incomplete structures, indicating that the three oligonucleotide molecules hybridized successfully to form the complete K^+^ DNA nanosensor, creating a larger overall structure (Fig. 3). There are multiple faint bands in the columns that contain hybridized structures, indicating that the three oligonucleotides did not hybridize with 100% efficiency (Fig. 3). However, the locations of the bright bands indicate that the majority of oligonucleotides formed a larger structure that affected its movement down the column, confirming the successful formation of the DNA nanosensor complex.

### Fluorescence spectrum measurement

**Fig. 4:**
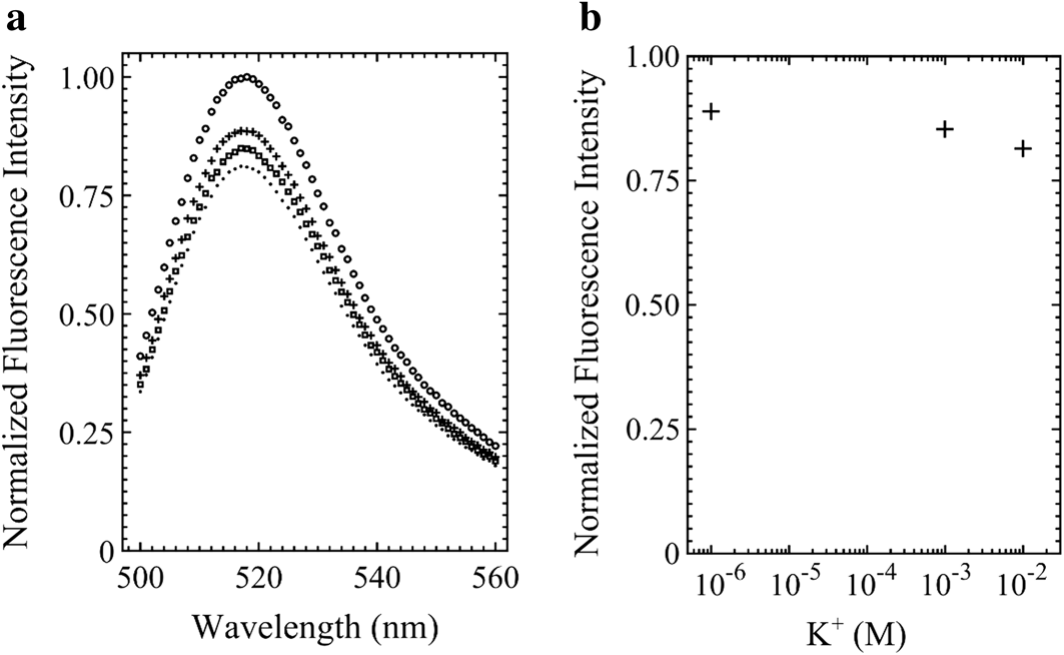
Normalized fluorescence intensity as a function of potassium concentration. **a** The fluorescence intensity of the nanosensor with a concentration of 1 µM was measured after adding 0 M (control), white circle, 1 µM, plus sign, 1 mM, square, and 10 mM, black circle, K^+^. **b** The peak normalized fluorescence intensity decreases with increased K^+^. PB + MgCl_2_ served as a control buffer

Once the formation of the DNA nanosensors was confirmed using the PAGE gel, the function of the nanosensors was tested using fluorescence spectrum assays with a fluorescence spectrometer. During these experiments, the fluorescence intensity was measured as a function of potassium concentration (Fig. 4a). To define a control fluorescence intensity level, fluorescence was measured in the presence of a control buffer (PB + MgCl_2_), without K^+^. Following the baseline measurement of the control buffer, K^+^ was added with concentrations of 1 µM, 1 mM, and 10 mM in different wells, each containing 1 μM DNA nanosensor solution. At the peak emission wavelength (518 nm), the normalized fluorescence intensity decreased by 11.1%, 14.7%, and 18.6%, respectively, for each concentration of K^+^ when compared to the baseline measurement without K^+^. While higher concentrations of potassium caused a greater decrease in fluorescence intensity (Fig. 4a), the decrease in the fluorescence signal is not proportional to the concentration of potassium added (Fig. 4b). The lack of proportionality in the fluorescence signal decrease upon addition of potassium demonstrates an intrinsic non-linearity in the system.

### DNA nanosensor concentration sensitivity

**Fig. 5:**
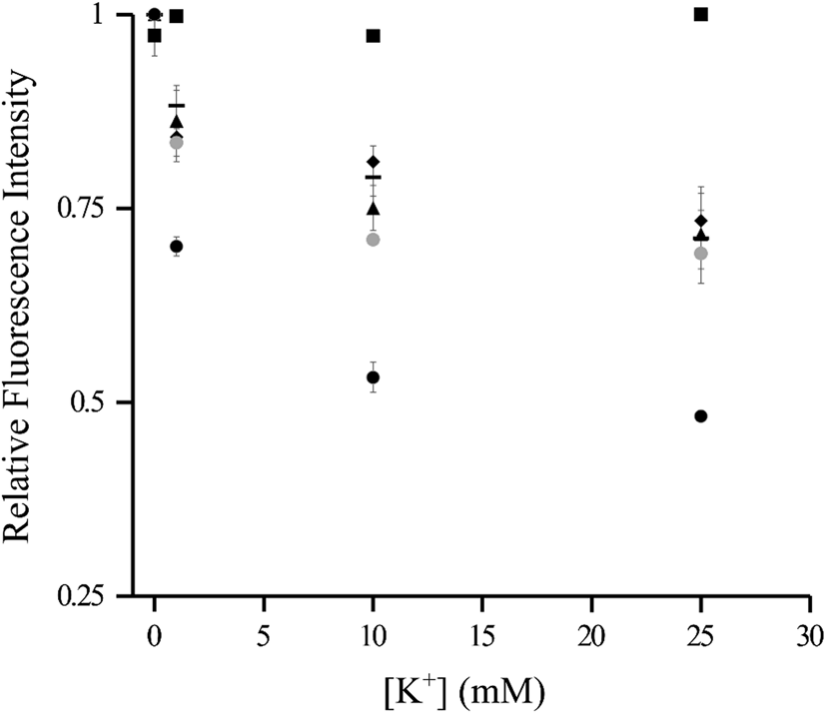
Relative fluorescence intensity response was measured as a function of potassium concentration. The relative fluorescence intensity was independently normalized with respect to the fluorescence signal of the corresponding DNA nanosensor concentration in the presence of the control buffer (PB + MgCl_2_), without K^+^. To optimize the concentration of DNA sensors, K^+^ was added at concentrations of 0, 1, 10, and 25 mM to DNA nanosensor samples of various concentrations: 0 µM (black square), 1 µM (figure dash), 5 µM (black diamond), 10 µM (black up-pointing triangle), 16.6 µM (gray circle), and 33.3 µM (black circle)

The function of the K^+^ DNA nanosensor was further tested using a fluorescence spectrometer in real time (Fig. 5). Various concentrations of the DNA nanosensor were tested to optimize the sensor response upon adding K^+^. To define a control fluorescence intensity level, fluorescence was measured in the presence of the control buffer (PB + MgCl_2_) without K^+^. No obvious changes in fluorescence were observed. Following the baseline control measurements, K^+^ was added serially to each sample to final concentrations of 1, 10, and 25 mM. Following the addition of K^+^, the fluorescence intensity signal was recorded after reaching steady state. The fluorescence signal for each DNA nanosensor concentration was normalized with respect to the fluorescence intensity of the corresponding nanosensor concentration in the control buffer (PB + MgCl_2_) without K^+^. A decrease in fluorescence was expected upon each individual addition of potassium since potassium binds to the DNA nanosensor leading to a conformational change that quenches the fluorescence signal. Once again, we observed a lack of proportionality in the change of the fluorescence level to the concentration of potassium added (Fig. 5), further demonstrating the non-linearity in the system. Increasing the DNA nanosensor concentration resulted in a larger relative signal decrease when K^+^ was added, suggesting that larger concentrations of the DNA nanosensor have a more sensitive response to K^+^ (Fig. 5).

### Immobilized DNA nanosensor concentration sensitivity

**Fig. 6:**
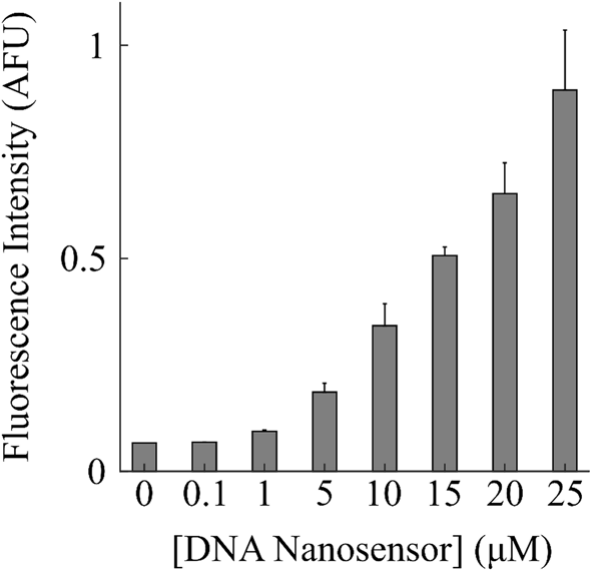
Fluorescence intensity measurements from DNA nanosensors coated on a glass slide at different concentrations of 0, 0.1, 1, 5, 10, 15, 20, and 25 µM

All measurements shown up to here demonstrated that DNA nanosensors in solution are sensitive to K^+^. Next, we wanted to determine if the sensitivity of the DNA nanosensor varied similarly when immobilized on a surface. DNA nanosensors were coated on a glass slide at varying concentrations that were relevant to the microfluidic device experiments. Figure 6 shows that at the 0 and 0.1 µM DNA nanosensor concentration, the fluorescence signal measured by the camera is due to background noise. However, as the concentration increased from 1 to 25 µM, the normalized fluorescence intensity increased nonlinearly with the largest increase of 24% occurring from 20 to 25 µM of DNA nanosensor.

### Microfluidic device experiment

**Fig. 7:**
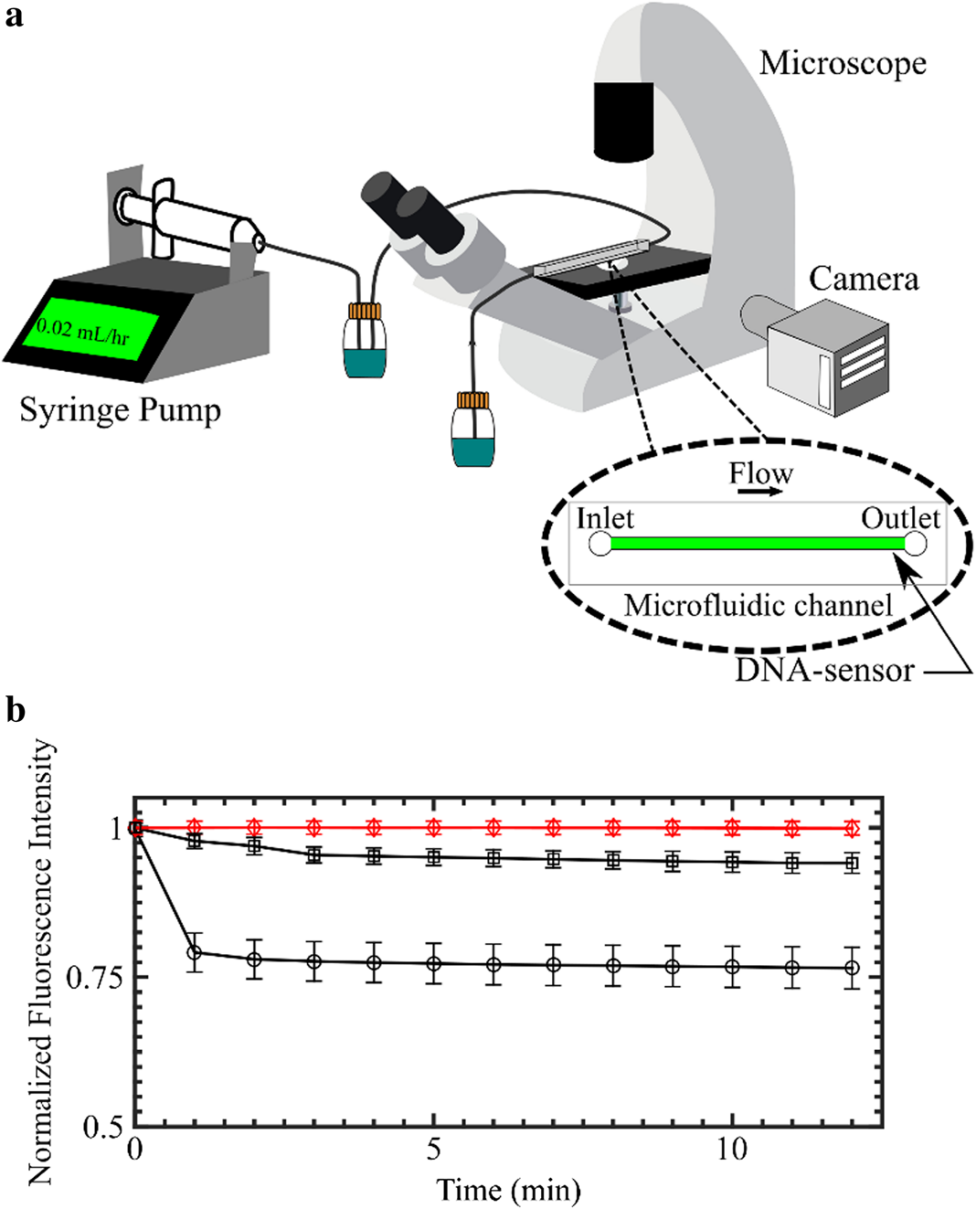
**a** DNA nanosensors with a concentration of 25 µM were immobilized on the bottom surface of a microfluidic device channel. The microfluidic device was infused with a syringe pump and imaged with microscope. **b** A PB + MgCl_2_ buffer (control) or KCl solution was run through the channel at 0.02 mL/h for 14 min. The normalized integrated fluorescence signal remained constant for the PB + MgCl_2_ buffer solution, lozenge, while the channels infused with 5 mM KCl, square, or 7 mM KCl, circle, decreased temporally

To further demonstrate the possibility of these K^+^ DNA nanosensors being incorporated into a point-of-care device, multiple channels in a microfluidic device were coated with the potassium DNA nanosensors (Fig. 7a). For a greater signal-to-noise ratio, the highest concentration solution of 25 µM was used for the coating of DNA nanosensors on the surface of the microfluidic channels. Channels were infused with either a control buffer solution (PB + MgCl_2_) or a potassium chloride (KCl) solution for at least 14 min. The fluorescence signal was monitored via periodic image acquisition using epifluorescence microscopy. After 14 min of infusion with the control buffer, no significant fluorescence signal change was observed (Fig. 7b). However, a significant decrease in fluorescence signal was observed when the K^+^ DNA nanosensors were exposed to 5 and 7 mM KCl in solution, which is within the physiological range of serum potassium (Fig. 7b). When the flow of 5 mM KCl contacted the K^+^ DNA nanosensors at the 1-min mark, the fluorescence signal decreased initially by 2.2%. Each minute following, the fluorescence signal decreased gradually, by percent decreases of 3.1, 4.5, and 4.7, respectively, before reaching a near-constant fluorescence intensity value after 4 min of infusion. In contrast, when the flow of 7 mM KCl contacted the K^+^ DNA nanosensors at the 1-min mark, the fluorescence signal decreased substantially by 20.8%. The fluorescence signal then decreased gradually, by percent decreases of 21.9, 22.2, and 22.5, respectively, before reaching a near-constant fluorescence intensity value. Although no major changes in fluorescence signal were observed after 4 min, the system was allowed to run for an additional 10 min to ensure attainment of a steady-state signal. These data demonstrate that bound DNA nanosensors can provide a spatial temporal signal of the presence of potassium in a solution at physiological concentrations. K^+^ was able to quench the fluorescence signal emitted by the DNA-based nanosensor over time in the device while the control buffer did not.

### DNA nanosensor ion selectivity measurement

**Fig. 8:**
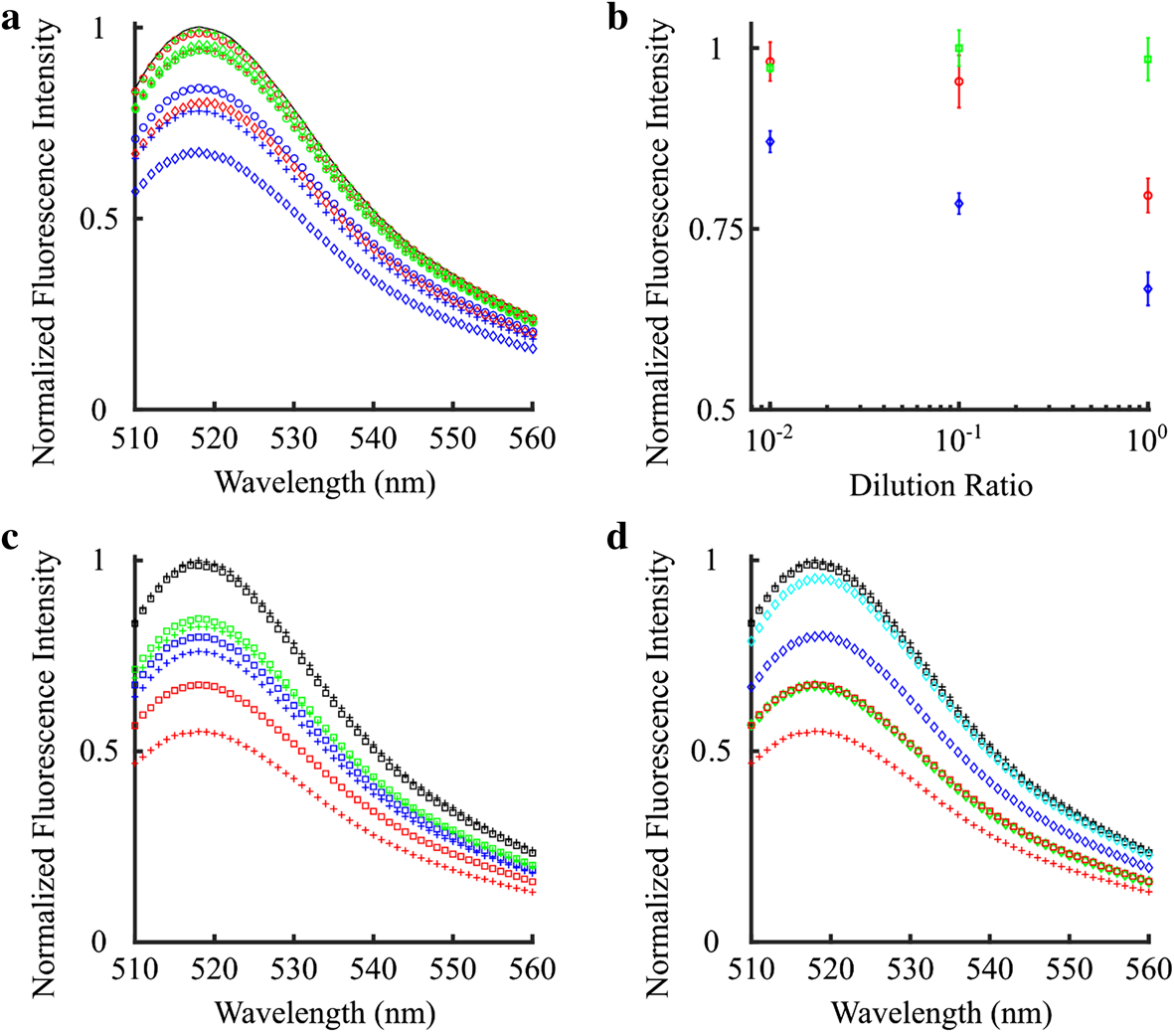
**a** DNA nanosensor selectivity for buffer solutions with no additional ions (black), figure dash 0 mM, Na^+^ (green), circle 1.4 mM, + 14 mM, lozenge 140 mM, Ca^2+^ (red), circle 0.024 mM, + 0.24 mM, lozenge 2.4 mM, and K^+^ (blue), circle 0.1 mM, + 1 mM, lozenge 10 mM. **b** DNA nanosensor selectivity at optimal wavelength (518 nm) for Na^+^ (green), square, Ca^2+^ (red), circle, and K^+^ (blue), lozenge, for three different dilutions 1×, 0.1×, and 0.01×. **c** DNA nanosensor selectivity for buffer solutions with no additional ions (black), and three different dilutions 0.01× (Na^+^ 1.4 mM, Ca^2+^ 0.024 mM, K^+^ 0.1 mM, green), 0.1× (Na^+^ 14 mM, Ca^2+^ 0.24 mM, K^+^ 1 mM, blue), and 1× (Na^+^ 140 mM, Ca^2+^ 2.4 mM, K^+^ 10 mM, red), with, square, or without, plus sign, EGTA. **d** DNA nanosensor selectivity for buffer solutions with no additional ions (black) plus sign 0 mM, Na^+^ (cyan) lozenge 140 mM, Ca^2+^ (blue) lozenge 2.4 mM, K^+^ (green) lozenge 10 mM, and all three ions (Na^+^ 140 mM, Ca^2+^ 2.4 mM, K^+^ 10 mM, red) plus sign without EGTA. EGTA is added to the buffer solutions with no additional ions (black) square 0 mM and all three ions (Na^+^ 140 mM, Ca^2+^ 2.4 mM, K^+^ 10 mM, red) square

DNA-based oligonucleotide nanosensors that can measure K^+^ using G-quadruplex can only serve as a viable alternative technology if sufficiently selective. The selectivity of the DNA nanosensor was tested in the presence and absence of Na^+^, Ca^2+^ or K^+^ for varying concentrations. First, the fluorescence intensity of the DNA nanosensors was measured in the absence of Na^+^, Ca^2+^ and K^+^ to establish a baseline (Fig. 8a). The normal concentration of Na^+^ in human blood is about 140 mM. This concentration of sodium decreased fluorescence intensity by about 4.8%, which was very similar to the 6.2% and 1.0% respective drops observed with 1.4 and 14 mM of Na^+^ (Fig. 8a, b). Changes in fluorescence intensity were quantified only at the peak intensity point, which coincided with the 518 nm wavelength. Similarly, Ca^2+^ ions at the physiological concentration of 2.4 mM, 0.24 mM and 0.024 mM decreased the fluorescence intensity by 20%, 5.9%, and 1.6%, respectively. The largest changes were observed when K^+^ was added. The addition of K^+^ at 10, 1.0 and 0.1 mM caused the fluorescence intensity to decrease by 32.8%, 22.1% and 16%, respectively. These results demonstrated that the DNA nanosensor was more selective for K^+^ than Na^+^, but still sufficiently selective for Ca^2+^ to affect the measurements.

To address the moderate selectivity of Ca^2+^ by the DNA nanosensors, the experiments from Fig. 8a were repeated by including or excluding EGTA, a calcium chelator. As a control, the DNA nanosensor fluorescence intensity was measured in the absence of Na^+^, Ca^2+^, and K^+^ ions, and the intensity did not change with the addition of EGTA (Fig. 8c). Diluting the physiologically relevant Na^+^, Ca^2+^, and K^+^ cation concentration by 100 times decreased fluorescence intensity by 17.4%, while adding EGTA to this cation concentration decreased the fluorescence signal by 15.2%. Although similar in trend, a slightly larger effect due to EGTA was observed when the cation concentration was diluted by ten times yielding a 23.8% and 20% decrease in fluorescence intensity without and with EGTA, respectively. However, the greatest effect due to EGTA was observed at physiologically relevant cation concentrations. Adding 140 mM Na^+^, 2.4 mM Ca^2+^, and 10 mM K^+^ decreased the fluorescence intensity by 44.8%, while the addition of EGTA yielded a smaller decrease of 32.6%.

Importantly, when adding Na^+^, Ca^2+^, or K^+^ independently at the corresponding physiological concentrations, the DNA nanosensor clearly shows greater selectivity for K^+^ (Fig. 8d). The presence of 10 mM K^+^ alone decreased the fluorescence intensity by 32.8%, while adding all three cations simultaneously, decreased the signal by 44.8%. However, addition of EGTA to the solution with all three cations at approximately physiological concentrations decreased the fluorescence signal only by 32.6%, completely abrogating the effects of Ca^2+^ as shown at the peak wavelength of 518 nm. The value of 32.6% with all three cations and EGTA is comparable to the 32.8% percent intensity decrease with K^+^ alone, demonstrating that the DNA nanosensor can be used for selective K^+^ detection in the presence of EGTA.

## Discussion

To the best of our knowledge, these are the first sets of experiments to demonstrate a simple proof-of-concept microfluidic device to measure potassium in a flowing solution using DNA-based G-quadruplex nanosensors. Low flow rate laminar flows were used to infuse the channels [28]. However, higher flow rates (data not shown) can be employed, yielding much faster fluorescence-quenching rates. In these experiments, a homogeneous solution was used. In a heterogeneous solution like blood, pulsatile flow with a retrograde component can be employed to promote mixing, ensuring that a uniform concentration of electrolytes interacts with the K^+^ DNA nanosensors.

These experiments were designed to demonstrate the validity of point-of-care potassium-measuring devices for potential future use at the point of care for hemodialysis patients. The concentrations of 5 mM and 7 mM KCl tested in the buffer solution match physiological potassium blood concentration levels, and are relevant to hemodialysis patients [13, 29, 30]. Future studies can be conducted to optimize the G-quadruplex potassium nanosensors to reduce the influence from serum molecules that may alter the signal and more accurately measure physiological potassium concentrations in whole blood. It has been previously demonstrated that an optimized G-quadruplex sequence can measure potassium selectively in blood, without interference from competitive ions at physiological concentrations [22]. To demonstrate selectivity, our study used EGTA to abrogate the influence of calcium, which is completely feasible for clinical practice since calcium chelators are routinely used to inhibit coagulation during blood draws [31].

The novelty in this research arises from being able to use a lab-on-a-chip device that, if mass produced, can potentially cost less than current techniques and can measure molecules in blood quickly with high accuracy and with less blood volume required [32]. In contrast, the current procedure is to draw blood from a patient once a month on average, send it to a laboratory and wait at least 24 h for the blood test results [33]. Although this novel technology has the potential of being less expensive, the most important benefit is that the hemodialysis session can be modified instantaneously for each patient, transforming traditional hemodialysis to personalized medical therapy, where the blood-filtering needs of each patient are fine-tuned similar to the physiological kidney function. If key electrolytes like potassium could be monitored more efficiently to provide an individual profile for each patient, treatments could be more personalized and complications such as sudden cardiac death may be decreased.

## Conclusions

We present proof-of-concept results for a microfluidic lab-on-a-chip device with external dimensions less than 2 cm that can quickly and accurately measure potassium using a minute amount of fluid. Our device utilizes novel DNA-based fluorescence oligonucleotide nanosensors to detect the presence of potassium flowing through a microfluidic channel, as an initial proof-of-concept for a lab-on-a-chip point-of-care device. After exposing the lab-on-a-chip nanosensor to potassium for approximately 4 min, a decrease in fluorescence signal was observed compared to control cases with buffer. Other commercially available devices require larger samples of blood and 24 h of turnaround time to receive results, a burden that can be avoided using a microfluidic device. This device would need further optimization to enhance uniform binding to a surface and improve sensitivity and selectivity before being considered in the clinics. Eventually, this device can be integrated with routine hemodialysis sessions to measure blood contents multiple times throughout a hemodialysis session, enabling protocol adjustment similar to a healthy kidney, which is presently unavailable. Sampling multiple drops of blood per session can reduce costs by limiting medical complications, avoid unnecessary venipuncture, and provide a real-time assessment of the patient’s health. This lab-on-a-chip device may allow hemodialysis centers to provide personalized medicine to patients, potentially reducing mortality rates and increasing quality of life.

## Data Availability

Data related to the current study are available from the corresponding author on reasonable request.
